# The World Federation of Hemophilia World Bleeding Disorders Registry: insights from the first 10,000 patients

**DOI:** 10.1016/j.rpth.2023.102264

**Published:** 2023-11-20

**Authors:** Donna Coffin, Emma Gouider, Barbara Konkle, Cedric Hermans, Catherine Lambert, Saliou Diop, Emily Ayoub, Ellia Tootoonchian, Toong Youttananukorn, Pamela Dakik, Ticiana Pereira, Alfonso Iorio, Glenn F. Pierce, World Bleeding Disorders Registry Participating Investigators, M. Abdel Mohsen, T. Adeyemo, G. Ai Sim, N. Al-Rahal, C. Alexis, T. Ali, O. Awodu, B. Aysarieva, A. Aziz, N. Barsallo, A. Biswas, A. Blair, J. Blatny, M. Borhany, D. Castillo, C. Catarino, A. Chuansumrit, M. Coetzee, A. Darwish Mohamad Ibrahim, S. Diop, A. Djenouni, A. El Ekiaby, M. El Khorassani, K. Fawcett, A. Ganieva, S. Govindan, D. Gwarzo, S. Hailemariam, P. Harper, T. Hassan, M. Hassan, C. Hermans, F. Hernandez, A. Imran, J. John, B. Keikhaei, T. Kotila, C. Liam, W. Marhaeni, D. Mbanya, P. Mekjarusgul, N. Meknassi, D. Micic, Y. Mlombe, R. Motusheva, D. Munube, A. Nagao, S. Najmi, V. Narayana Pillai, T. Narbekov, D. Nasution, R. Natesirinilkul, L. Nchimba, M. N’dogomo, D. Neme, P. Nguyen, HM. Nguyen, M. Nguyen Thi, RK. Nigam, F. Njuguna, T. Nwagha, A. Obeida, S. Owusu-Ofori, J. Palascak, G. Pellegrini, C. Philip, CL. Ping, B. Poudyal, G. Rabbani, OA. Rakoto Alson, H. Razali, T. Ruchutrakul, A. Ruiz-Saez, S. Saengboon, N. Salhi, M. Satti, T. See Guan, S. Shah, T. Shikuku, N. Si Yuan, N. Sidarthan, T. Siew Looi, N. Songthawee, D. Sosothikul, P. Surapolchai, S. Suryani, NA. Syakira, A. Thevarajah, TJ. Tzong, C. Udo, L. Wong, S. Yuguda, T. Zafar, M. Zaman Miah

**Affiliations:** 1 World Federation of Hemophilia, Montreal, Quebec, Canada; 2 Service d’hématologie biologique Hemophilia Center Aziza Othmana, Faculté de Médecine de Tunis, Tunis, Tunisia; 3 Washington Center for Bleeding Disorders, Bloodworks Northwest, Seattle, Washington, USA; 4 Department of Internal Medicine, Université Catholique de Louvain, Louvain-la-Neuve, Belgium; 5 Haemostasis and Thrombosis Unit, Division of Hematology, Cliniques Universitaires Saint-Luc, Brussels, Belgium; 6 Department of Hematology, National Blood Transfusion Center, University Cheikh Anta Diop, Dakar, Senegal; 7 Department of Clinical Epidemiology and Biostatistics, McMaster University, Hamilton, Ontario, Canada; 8 Department of Medicine, McMaster University, Hamilton, Ontario, Canada

**Keywords:** bleeding disorders, global, hemophilia, registries

## Abstract

**Background:**

The prevalence of hemophilia varies globally, with close to 100% of patients diagnosed in high-income countries and as low as 12% diagnosed in lower-income countries. These inequalities in the care of people with hemophilia exist across various care indicators.

**Objectives:**

This analysis aims to describe the clinical care outcomes of patients in the World Bleeding Disorders Registry (WBDR).

**Methods:**

In 2018, the World Federation of Hemophilia developed a global registry, the WBDR, to permit hemophilia treatment centers to collect clinical data, monitor patient care longitudinally, and identify gaps in management and treatment.

**Results:**

As of July 18, 2022, 10,276 people with hemophilia were enrolled from 87 hemophilia treatment centers in 40 countries. Nearly half (49%, *n* = 5084) of patients had severe hemophilia; 99% were male, 85% had hemophilia A, and 67% were from low-middle-income countries. Globally, the age of diagnosis for people with severe hemophilia has improved considerably over the last 50 years, from 82 months (∼7 years) for those born before 1980 to 11 months for those born after 2010, and most prominently, among people with severe hemophilia in low- and low-middle-income countries, the age of diagnosis improved from 418 months (∼35 years) for those born before 1970 to 12 months for those born after 2010. Overall, the age of diagnosis of people with hemophilia in low- and low-middle-income countries is delayed by 3 decades compared to patients in upper-middle-income countries and by 4 decades compared to patients in high-income countries.

**Conclusion:**

Data reveal large treatment and care disparities between socioeconomic groups, showing improvements when prophylaxis is initiated to prevent bleeding. Overall, care provided in low-income countries lags behind high-income countries by up to 40 years. Limitations in the interpretation of data include risk of survival and selection bias.

## Essentials

• Analysis of outcomes of patients enrolled in the World Bleeding Disorders Registry.
• Eighty-seven hemophilia treatment centers around the world participate in the World Bleeding Disorders Registry.
• Data on 10,276 people with hemophilia demonstrate wide discrepancies in care globally.
• Care provided in low-income countries lags behind high-income countries by up to 40 years.

## 1 Introduction

Hemophilia A and B, caused by variants in the *F8* and *F9* genes, respectively, are rare X-linked monogenic diseases characterized by low or absent circulating levels of coagulation factors VIII and IX. These result in repeated bleeding, which can lead to long-term disability and, if untreated, premature death [1]. The prevalence of hemophilia is estimated to be 1 in 5000 males, and the incidence is estimated to be 1 in 1333 live male births [2]. Disease severity is determined by the residual factor activity in the patient’s blood. People with mild hemophilia retain 5% to 40% of normal factor activity in their blood, and bleeding will mostly occur after major trauma or surgery. People with moderate hemophilia retain 1% to <5% of factor activity, leading to occasional spontaneous bleeding episodes or prolonged bleeding triggered by trauma or surgery. Severe hemophilia manifests clinically with spontaneous bleeding episodes into the joints or muscles with <1% of factor activity retention [3]. Left untreated, people with severe hemophilia experience frequent bleeding episodes, arthropathy, reduced quality of life, and shortened life expectancy.

The probability of people with hemophilia receiving accurate diagnosis as well as access to treatment and care is highly correlated with the economy of the country in which they live [4,5]. In high-income countries (HICs), close to 100% of patients are identified, while in some low-income countries (LICs), less than 12% of patients are identified [4]. The unidentified patients remain undiagnosed and can die prematurely due to the lack of diagnosis and timely treatment. One factor contributing to the challenge of identifying and managing people with hemophilia in LIC is the lack of systematic infrastructure to document clinical care over the long term and evaluate health outcomes. Most HICs, on the other hand, have established national patient registries to collect longitudinal data on people with hemophilia and, in some cases, use them to monitor patient outcomes, leading to improved clinical care [6].

Patient registries are an important source of real-world data that offer accessible information based on large patient sample sizes followed longitudinally [7]. Registries are also an excellent source of data to address questions that are typically not evaluated in randomized clinical trials, including how clinical settings, care models, and health system characteristics influence treatment outcomes. Given the value of real-world data and the increasing demand for evidence in hemophilia care, the World Federation of Hemophilia (WFH) launched the World Bleeding Disorders Registry (WBDR) [8], a global registry to collect data on people with hemophilia. This article describes the development and data collection methods of the WBDR and reports on changes in clinical care outcomes over time, including diagnostic capacity, access to prophylaxis, annual bleed rate (ABR), and how work productivity is affected in people with severe hemophilia.

## 2 Methods

### 2.1 Design

The WBDR is an observational, longitudinal, prospective, global registry of people with hemophilia [8]. The WFH serves as the coordinating center for data collection, cleaning, and analysis. The WBDR data are collected through a web-based portal, which contains the electronic case report forms. The WBDR data are de-identified and maintained on a secure registry platform, which is compliant with the European Union’s Conformité Européenne marking regulation on medical devices, Directive 93/42/EEC, the United Kingdom's Information Governance Toolkit standard, and the General Data Protection Regulation (https://wfh.org/data-collection/#wbdr). Only authorized hemophilia treatment center (HTC) staff have access to the password-protected platform, and each HTC has access to their patient data only and cannot view patient data from any other HTC.

The study population, recruitment processes, data collection, and analyses are described below.

### 2.2 Study population

#### 2.2.1 Recruitment of HTCs

HTCs from around the world are eligible to participate in the WBDR if they obtain ethics approval from their local organization. Once the HTC has obtained ethics approval, it will gain access to the web-based data entry system. HTCs are encouraged to invite all consecutive people with hemophilia receiving care at their HTC to participate in the registry.

#### 2.2.2 Recruitment of people with hemophilia

All individuals diagnosed with hemophilia A, B, or of an unknown type who are followed at participating HTCs are eligible for enrollment in the WBDR. Individual patients are recruited by HTC staff and are required to provide informed consent. There are no exclusion criteria.

Identification of potential HTCs is supported by WFH’s in-country regional managers and WFH-affiliated national member organizations in 147 countries. As part of WFH’s regional strategy, countries are provided with the training and tools needed to progress through the 6 pillars of development, which comprise the WFH comprehensive development model [9]. HTCs in countries with data collection capacity and without a national patient registry were targeted for a first round of invitations, with an aim for regional and economic representation. Within each country, HTCs were prioritized for participation in the WBDR based on their data collection capacity. In addition to direct invitations, international conferences and regional workshops and meetings were used as venues to recruit HTCs for participation in the WBDR. Countries with existing, robust national hemophilia registries were considered for a country-wide strategy of direct data transfer from their registry to the WBDR through an International Data Integration Program. HTCs are required to obtain ethics approval or exemption from their local institutional review boards. All supporting documents are provided in local languages by the WFH along with training on patient recruitment, obtaining informed consent, and data entry.

#### 2.2.3 Datasets

The WBDR was launched in January 2018 with a data set consisting of patient demographics, hemophilia-related treatments and bleeding events, and comorbidities collected at baseline and each subsequent follow-up visit (Supplementary Table S1). Validated instruments to measure quality of life (EQ-5D-5L) [10], burden of disease (Patient-Reported Outcomes Burdens and Experiences) [11], and functional status as well as a COVID-19 module were added in 2020. Data is collected retrospectively for the 6 months prior to entering the WBDR and then prospectively at each clinic visit thereafter. All data in the core data set undergo a systematic data cleaning process on 100% of the data fields. This includes automated edit checks at the data entry level and an automated and auditable process of assessing the entry of the data field against its expected logic, format, and range. Data discrepancies are resolved through data clarification forms. The WBDR database is available in English, French, Spanish, and Russian and has recently been expanded to include patients with von Willebrand disease in 2023.

### 2.3 Statistical analysis

Data from all participants enrolled in the WBDR since its launch on January 26, 2018, to July 18, 2022, were analyzed. Descriptive statistics were used to report demographic data on HTCs and participants in the WBDR. In this analysis, only people with severe hemophilia were included (*N* = 5084) (as this is the most reliably diagnosed and enrolled hemophilia type) and reported with frequency or median and IQRs. Outcome data were stratified by the decade in which the participant was born (birth cohort). A total of 6 birth cohorts were used, from 1939 to 2020, each spanning a decade, except for the first birth cohort, where 3 decades were combined (1939 to 1970) due to low patient numbers (*N* = 239 born over the 3 decades). The data were also analyzed by gross national income (GNI) as categorized by the World Bank Group [12]. In 2020, the GNI ranges in US dollars were defined as follows: low income, $0 to $1045; lower middle income, $1046 to $4095; upper middle income, $4096 to $12,695; and high income, $12,695 or more. For this analysis, GNI categories of low and lower middle income were merged due to the small sample size in the low-income category (*N* = 153). No additional confounding was considered.

Four indicators of care were selected for analysis: age of diagnosis, prophylaxis use, ABR, and work productivity. Participants were included in the age-at-diagnosis analysis if both a date of birth and date of diagnosis were reported in the WBDR. Prophylaxis use and ABR were calculated on 1 year of data. The year 2020 was chosen as it was the most recent year with full audited data in the WBDR. The population analyzed for prophylaxis and ABR included severe patients enrolled until the end of 2020, which resulted in an initial sample size of 3657 participants. Among these, ABR analyses included only severe patients with at least 1 visit reported in 2020. Participants were considered to receive prophylaxis in 2020 if they had at least 1 record of prophylaxis use in 2020, including ongoing prophylaxis treatments initiated prior to 2020. ABR was calculated as the number of bleeding events reported in 2020, divided by the number of months in the reporting period (the minimum reporting period was 30 days for inclusion) and multiplied by 12. The population analyzed for work productivity included all people with severe hemophilia aged >20 years, with data on work productivity reported in the WBDR. The last 2 birth cohorts (2001-2010 and 2011-2020) were not included in the analysis of work productivity as patients born in these 2 decades would not be of working age. The sample size for this analysis was 1621 people with hemophilia.

## 3 Results

**Figure 1:**
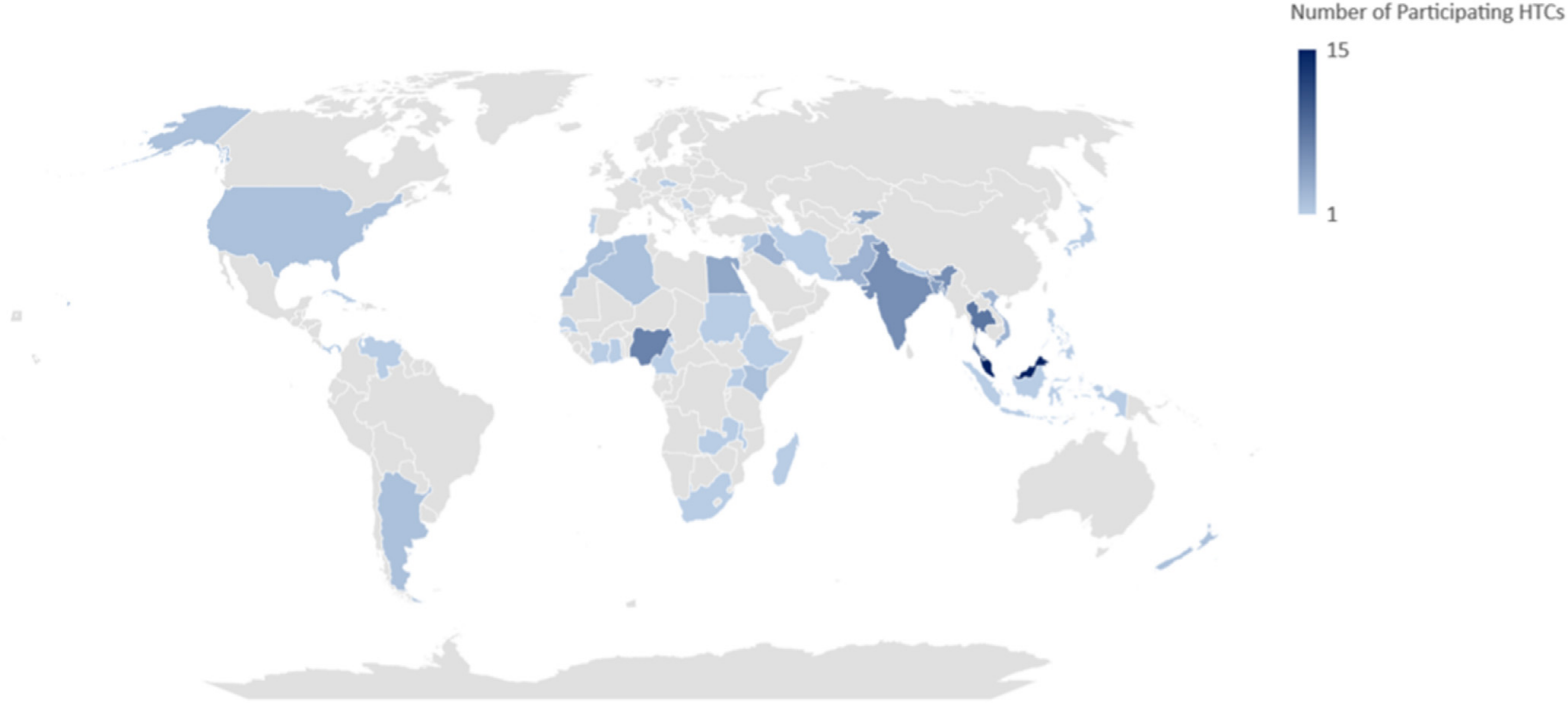
World map highlighting countries with participating hemophilia treatment centers (HTCs).

As of July 18, 2022, 10,276 people with hemophilia were enrolled in the WBDR from 87 HTCs in 40 countries (Figure 1, Supplementary Table S2). Of these, 5084 had severe hemophilia (49%). The majority of those enrolled were male (99%), with hemophilia A (85%), and from low-middle-income countries (LMICs) (67%) (Table 1).

**Table 1:** Demographic variables of people with hemophilia enrolled in the World Bleeding Disorders Registry.

Demographics of people with hemophilia					
Demographic variable	Mild (*n* = 1704)	Moderate (*n* = 3154)	Severe (*n* = 5084)	Unknown (*n* = 334)	Total (*N* = 10,276)
Hemophilia type, *n* (%)					
A	1427 (84)	2582 (82)	4435 (87)	243 (73)	8687 (85)
B	271 (16)	566 (18)	645 (13)	53 (16)	1535 (15)
Unknown	6 (0)	6 (0)	4 (0)	38 (11)	54 (1)
Sex,^a^*n* (%)					
Female	31 (2)	18 (1)	23 (0)	10 (3)	82 (1)
Male	1673 (98)	3135 (99)	5061 (100)	324 (97)	10,193 (99)
Age					
Median (Q1, Q3)	25 (14, 39)	20 (11, 30)	20 (11, 33)	16 (9, 26)	21 (12, 33)
Region, *n* (%)					
Africa	97 (6)	253 (8)	552 (11)	261 (78)	1163 (11)
Americas	56 (3)	46 (1)	289 (6)	6 (2)	397 (4)
Eastern Mediterranean	346 (20)	919 (29)	1437 (28)	35 (10)	2737 (27)
Europe	414 (24)	163 (5)	389 (8)	11 (3)	977 (10)
Southeast Asia	561 (33)	1347 (43)	1274 (25)	17 (5)	3199 (31)
Western Pacific	230 (13)	426 (14)	1143 (22)	4 (1)	1803 (18)
GNI,^a^*n* (%)					
HIC	437 (26)	154 (5)	499 (10)	7 (2)	1097 (11)
UMIC	279 (16)	338 (11)	1207 (24)	4 (1)	1828 (18)
LMIC	892 (52)	2514 (80)	3225 (63)	250 (75)	6881 (67)
LIC	96 (6)	148 (5)	153 (3)	73 (22)	470 (5)

GNI, gross national income; HIC, high-income country ($12,695 or more); LIC, low-income country ($0-$1045); LMIC, lower-middle-income country ($1046-$4095); UMIC, upper-middle-income country ($4096-$12,695).

a One participant defined themselves as “other” sex and is not included in this table.

### 3.1 Indicator of care of hemophilia: age at diagnosis

**Figure 2:**
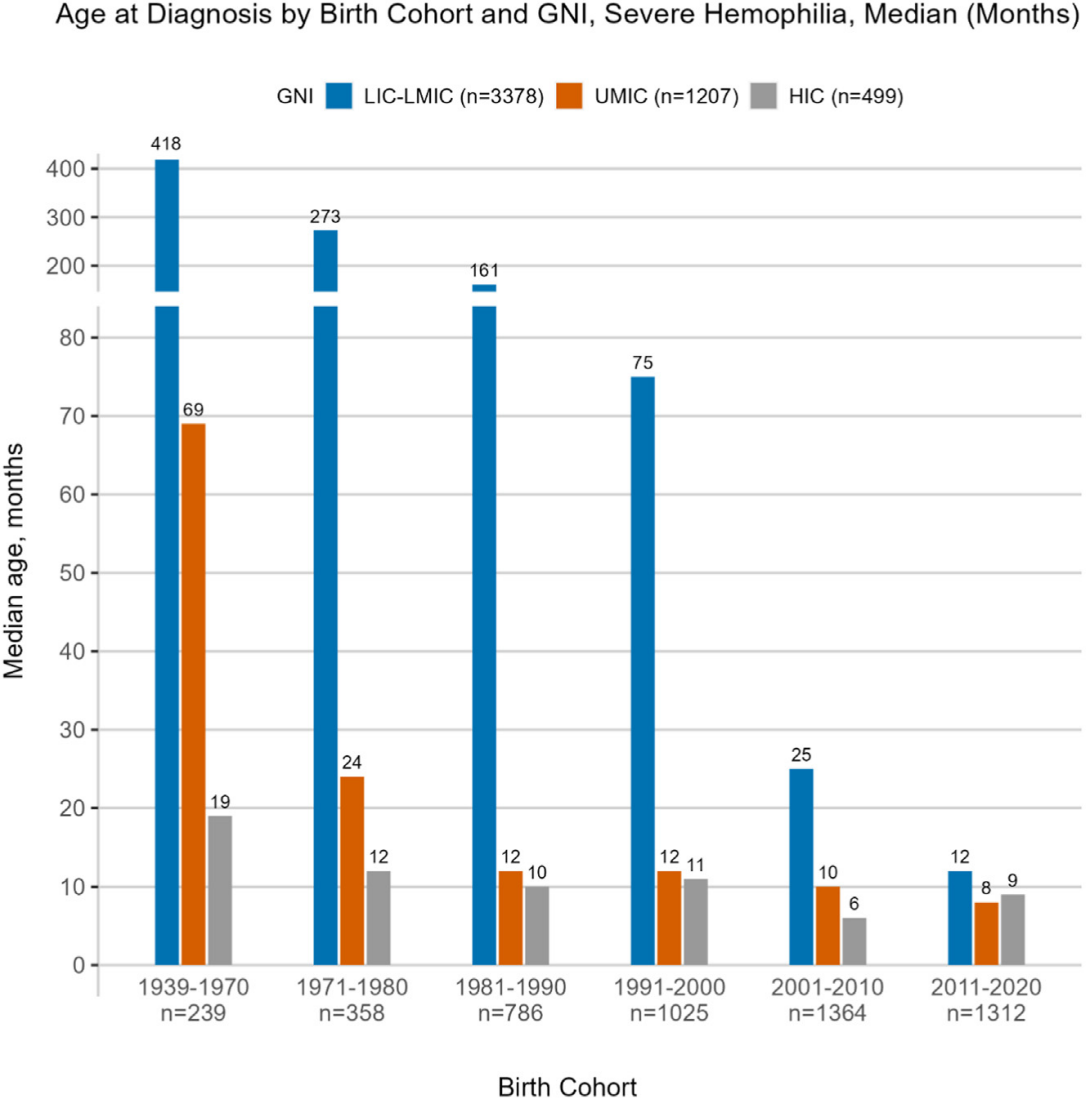
Age at diagnosis of people with severe hemophilia by birth cohort and gross national income (GNI). Age (in months) is expressed as median with IQR. HIC, high-income country ($12,695 or more); LIC, low-income country ($0-$1045); LMIC, lower-middle-income country ($1046-$4095); UMIC, upper-middle-income country ($4096-$12,695).

Table 2 and Figure 2 illustrate the age of diagnosis for people with severe hemophilia by birth cohort and GNI. Data on age of diagnosis were available for all people with severe hemophilia in the WBDR. Globally, the age of hemophilia diagnosis for people with severe hemophilia has improved considerably over the last 50 years, down from 82 months (∼7 years of age) for those born before 1980 to 11 months for those born more recently (after 2010). Severe patients in LICs and LMICs are currently being diagnosed at a median of 12 months, which is a considerable improvement from 418 months (∼35 years) for patients born before 1970. Patients born between 2011 and 2020 in upper-middle-income countries (UMICs) and HICs were diagnosed at 8 and 9 months, respectively. This is a considerable improvement compared to patients born prior to 1971 in UMICs and HICs, who were diagnosed at 69 and 19 months, respectively.

**Table 2:** Age at diagnosis of people with severe hemophilia by birth cohort and gross national income.

Birth cohorts	LIC-LMIC (*n* = 3378)	UMIC (*n* = 1207)	HIC (*n* = 499)	Total (*n* = 5084)
1939-1970	418 (240-577)	69 (15-201)	19 (7-36)	73 (16-418)
1971-1980	273 (84-421)	24 (6-119)	12 (3-31)	82 (12-297)
1981-1990	161 (30-280)	12 (6-72)	10 (3-24)	54 (11-220)
1991-2000	75 (15-180)	12 (6-39)	11 (3-20)	31 (9-135)
2001-2010	25 (8-89)	10 (6-25)	6 (0-14)	18 (7-64)
2011-2020	12 (6-31)	8 (5-12)	9 (3-14)	11 (6-24)
**Total**	**33 (9-128)**	**11 (6-36)**	**10 (3-21)**	**20 (7-88)**

Data are reported in months as median (IQR).

GNI, gross national income; HIC, high-income country ($12,695 or more); LIC, low-income country ($0-$1045); LMIC, lower-middle-income country ($1046-$4095); UMIC, upper-middle-income country ($4096-$12,695).

### 3.2 Prophylaxis use in the year 2020

**Figure 3:**
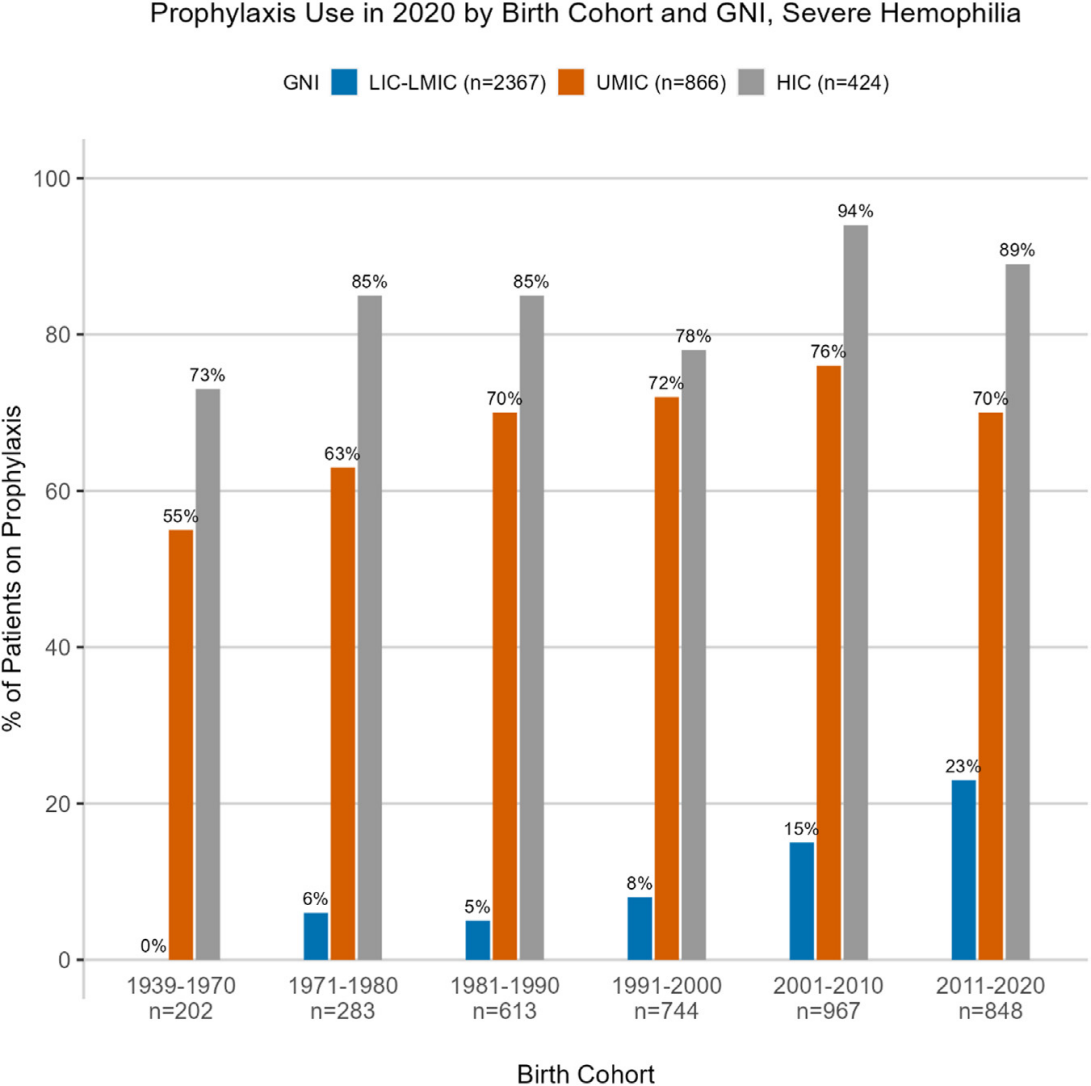
Prophylaxis use in people with severe hemophilia in 2020 by birth cohort and gross national income (GNI) 2020. HIC, high-income country ($12,695 or more); LIC, low-income country ($0-$1045); LMIC, lower-middle-income country ($1046-$4095); UMIC, upper-middle-income country ($4096-$12,695).

Table 3 summarizes the proportion of people with severe hemophilia receiving prophylaxis in 2020 by birth cohort and GNI. Data on prophylaxis use in 2020 were available for the 3657 people with severe hemophilia enrolled in the WBDR as of the end of 2020. Globally, 35% (*n* = 1277) of severe patients reported receiving prophylaxis in 2020, ranging from 13% in LICs and LMICs to 71% in UMICs to 84% in HICs (Table 3, Figure 3). In general, prophylaxis use in 2020 increased with each successively younger cohort. Patients aged 0 to 9 years in 2020 (birth cohort 2011-2020) were more likely to be on prophylaxis in 2020 compared to their older counterparts. The same is true for each successively higher GNI category.

**Table 3:** Prophylaxis use in people with severe hemophilia in 2020 by birth cohort and gross national income: 2020.

Birth cohorts	Age of patients in 2020 (y)	LIC-LMIC (*n* = 2367)	UMIC (*n* = 866)	HIC (*n* = 424)	Total (*N* = 3657)
1939-1970	50+	0 (0)	22 (55)	57 (73)	79 (39)
1971-1980	40-49	8 (6)	50 (63)	53 (85)	111 (39)
1981-1990	30-39	18 (5)	126 (70)	63 (85)	207 (34)
1991-2000	20-29	36 (8)	145 (72)	53 (78)	234 (31)
2001-2010	10-19	102 (15)	173 (76)	67 (94)	342 (35)
2011-2020	0-9	145 (23)	96 (70)	63 (89)	304 (36)
Total		309 (13)	612 (71)	356 (84)	1277 (35)

Data are reported as *n* (%).

HIC, high-income country ($12,695 or more); LIC, low-income country ($0-$1045); LMIC, lower-middle-income country ($1046-$4095); UMIC, upper-middle-income country ($4096-$12,695).

### 3.3 ABR in the year 2020

Table 4 summarizes the ABR of people with severe hemophilia in the year 2020 by birth cohort and GNI. Data on ABR reported in 2020 were available for 2513 people with severe hemophilia (69% of the severe patients enrolled in the WBDR as of the end of 2020 who met the criteria for inclusion in the ABR calculation). Globally, the median ABR was 4, ranging from 6 in LICs and LMICs to 2 in UMICs to 1 in HIC. In the HIC and UMIC, ABR tended to remain stable across all age cohorts. However, in LICs and LMICs, ABR was highest among younger patients and decreased with successively older patients (from an ABR of 6 among those aged 0-9 years [birth cohort 2011-2020] to an ABR of 4 among those aged >30 years [birth before 1991]).

**Table 4:** Annual bleed rate in people with severe hemophilia in 2020 by birth cohort and gross national income.

ABR					
People with hemophilia by birth cohort	Age of patients in 2020 (y)	LIC-LMIC (*n* = 1770)	UMIC (*n* = 383)	HIC (*n* = 360)	Total (*N* = 2513)
1939-1970	50+	4 (4-8)	1 (0-2)	0 (0-3)	3 (0-6)
1971-1980	40-49	4 (2-8)	1 (0-4)	0 (0-3)	2 (0-6)
1981-1990	30-39	4 (2-8)	1 (0-5)	1 (0-2)	4 (0-7)
1991-2000	20-29	6 (2-10)	2 (0-6)	0 (0-2)	4 (1-9)
2001-2010	10-19	6 (2-12)	1 (0-4)	1 (0-2)	4 (2-11)
2011-2020	0-9	6 (2-14)	2 (0-4)	0 (0-2)	4 (1-11)
**Total**		6 (2-11)	2 (0-5)	1 (0-2)	4 (1-9)

Data are reported as median (IQR).

ARB, annual bleed rate; HIC, high-income country ($12,695 or more); LIC, low-income country ($0-$1045); LMIC, lower-middle-income country ($1046-$4095); UMIC, upper-middle-income country ($4096-$12,695).

### 3.4 Impact of hemophilia on productivity

Table 5 summarizes the proportion of people with severe hemophilia who reported decreased work productivity. Participants were asked to report the effect hemophilia has had on their work status (eg, part-time work, long-term sick leave, not employed, or retired early due to hemophilia). Data were available on 1621 (67%) working-age people with severe hemophilia in the WBDR. Overall, 24% (387) reported decreased work productivity due to their hemophilia. In general, the impact of hemophilia on productivity decreased with each successively younger cohort and with each successively higher GNI category (28%, 14%, and 13% for LICs and LMICs, UMICs, and HICs, respectively).

**Table 5:** Reduced work productivity due to hemophilia in people with severe hemophilia by gross national income.

Reduced work productivity				
Patients by birth cohort	LIC-LMIC (*n* = 1172)	UMIC (*n* = 315)	HIC (*n* = 134)	Total (*n* = 1621)
1939-1970	36 (41)	4 (15)	9 (23)	49 (32)
1971-1980	44 (30)	7 (14)	6 (17)	57 (24)
1981-1990	117 (30)	20 (17)	3 (9)	140 (26)
1991-2000	127 (23)	14 (11)	0 (0)	141 (20)
Total	324 (28)	45 (14)	18 (13)	387 (24)

Data are reported as *n* (%).

HIC, high-income country ($12,695 or more); LIC, low-income country ($0-$1045); LMIC, lower-middle-income country ($1046-$4095); UMIC, upper-middle-income country ($4096-$12,695).

## 4 Discussion

The WBDR is the only global registry for people with hemophilia spanning several continents, which is unique in the area of rare diseases. The vast geographical and income-level representation of the WBDR makes it an ideal platform to compare standards of care around the world. Having standardized and harmonious data on patients from diverse countries allows for a more comprehensive analysis and comparison of data.

The data on age at diagnosis for people with hemophilia demonstrates an improved capacity for early diagnosis of patients over time; however, large discrepancies in diagnostic capacity remain between lower- and higher-income countries. Patients enrolled in the WBDR from LICs and LMICs are currently being diagnosed at a similar age as patients in HICs were being diagnosed in the 1970s and as patients in UMIC in the 1980s and 1990s, meaning people with hemophilia in LMICs are lagging behind patients in UMICs by 3 decades and behind patients in HICs by 4 decades in age of diagnosis. Given that the mainstay of therapy, and what is recommended in the WFH treatment guidelines [3], is prophylaxis starting by age 2 years, early diagnosis becomes a critical component in providing the best care for patients. Today, the capacity to diagnose in UMICs has improved to the same level as that in HICs despite being substantially lower before 1970. Lessons on how UMICs improved their diagnostic capacity could be applied to LICs and LMICs. Diagnostic and care delivery challenges in LICs and LMICs around the world are well documented and are being addressed through ongoing educational, training, and capacity-building programs delivered by the WFH and other bleeding disorders organizations around the world [[13], [14], [15], [16], [17], [18]]. Moreover, the WFH Humanitarian Aid program provides much-needed support to national member organizations, HTCs, and healthcare practitioners in emerging countries. This support includes donated factor and nonfactor replacement therapy as well as training and education related to treatment products. The expansion of the WFH Humanitarian Aid program in 2016, largely in LICs/LMICs and in younger patients, provides impetus for making early diagnoses since patients in these countries can now have easier access to treatment [19,20]. The impact of the increase in access to prophylaxis via the WFH Humanitarian Aid program can be seen in the younger cohorts (2001-2010 and 2011-2020) in LMICs and LICs, which increased to 15% and 23%, respectively.

Coupled with early diagnosis, early start of prophylaxis for people with severe hemophilia is critical to avoid long-term sequelae [3]. In a reduced capacity setting, where hemophilia treatment is scarce, countries often prioritize children for early prophylaxis use, who will benefit the most [14,15,17,20,21]. These data support this premise, showing that younger patients are more likely to be on prophylaxis in the current era compared to their older counterparts. However, overall, the global proportion of people with hemophilia on prophylaxis (35%) remains very low, indicating that countries, particularly lower-income countries, are not able to provide prophylaxis to all of their patients as recommended in the current treatment guidelines. This is supported by the Report on the WFH Annual Global Survey, which demonstrates that suboptimal treatment is common, particularly in LICs [4,5]. According to the 2021 Annual Global Survey, the median FVIII use globally is 2.5 IU per capita, which varies by GNI from 5.38 IU in HICs to 0.05 IU per capita in LICs [4]. Global strategies for improved access to prophylaxis are needed to avoid higher annual bleeds and subsequent disabling arthropathies. Paradoxically, our data show lower or stable bleed rates for older patients, who are also less likely to be on prophylaxis, although one would expect lower bleed rates in the context of higher prophylaxis use. This finding may be because bleed rates do decrease naturally among older patients, in part due to lack of mobility, and the level of prophylaxis use in the data (35% overall, 13% in LICs and LMICs) may simply not be high enough to demonstrate an observable impact on the global ABR. Additionally, many lower-resource countries rely on low-dose prophylaxis, which does confer reduced bleeding episodes, but the impact of low-dose prophylaxis on bleeding is not as beneficial as the impact of high-dose prophylaxis [22,23].

In people with severe hemophilia, the combination of late diagnosis, lack of prophylaxis, and high bleeding rate can have a negative impact on work productivity. Patients with a late diagnosis and lower access to prophylaxis have the biggest impact on reduced work productivity, namely patients from LICs and LMICs, with older cohorts fairing worse than younger cohorts.

### 4.1 Limitations

This analysis has limitations that should be considered, including selection bias, survival bias, the impact of disparities in access to healthcare services (which include differences in diagnostic capacity, availability of treatment, and ease of accessing treatment centers), the potential impact of COVID-19, and low patient numbers in certain categories.

The observational nature of registries poses an inherent risk of certain types of bias, particularly selection bias [24,25]. Selection bias arises when patients with certain characteristics are systematically more likely to be included in a registry, threatening the validity of the registry data. To minimize the effects of selection bias, participating HTCs are encouraged to invite all consecutive people with hemophilia who visit their center, thus limiting the possibility of selecting participants with specific disease characteristics. Not all HTCs have enrolled all of their patients into the WBDR; thus, the data are at risk of selection bias. In an attempt to quantify the potential risk of bias, we examined WBDR enrollment percentages per HTC. Data available for 54 of the 87 WBDR HTCs (62%) showed that an average of 54% of their patients had been enrolled in the WBDR, indicating that the data in the WBDR are not free of risk of selection bias. Over time, HTCs will continue to enroll their patients in the registry, aiming for closer to 100%, thus reducing the risk of selection bias; however, for now, interpretation of the data must allow for possible bias. Similarly, selection bias may occur at the HTC level. HTCs in the WBDR volunteer to participate and may not be representative of all HTCs in a region or country.

Survival bias also poses a risk in our observational study. Infant mortality rates are highest in LICs and LMICs, potentially leading to survival bias and a skewed or “healthier” subset of participants from LICs and LMICs in the WBDR [26]. Premature deaths disproportionately affecting people with severe hemophilia in LICs and LMICs may have skewed outcome comparisons (bleeding events, age of diagnosis) between income categories. LICs and LMICs may have reported fewer bleeds and older age at diagnosis than expected if nonsurvivors were included.

Differences in the impact of COVID-19 across birth cohorts may have introduced uncontrolled confounding, leading to a skewed relationship between patient age and the measured outcomes. For instance, during the pandemic, older patients might have faced more challenges in accessing treatment centers compared to their younger counterparts. These difficulties in access could have had adverse effects on the use of prophylaxis and the reporting of prophylaxis usage and bleeding data, potentially distorting the associations between the birth cohort and the study outcomes.

Several data classification aspects should be considered when interpreting the results. Country economic classifications can change annually. For this analysis, GNI categories from 2020 were used. Although some countries may have changed categories between 1939 and 2020, 2020 was deemed most suitable for this study. ABR and prophylaxis data were also based on 2020, the most recent audited year of data. However, the last cohort (2011-2020) included a few participants born in 2020 who may not have contributed a full year of data for ABR and prophylaxis for 2020. However, given the small number of participants (*n* = 5, 0.1%), the impact on results is expected to be minimal. The ABR and prophylaxis analyses were based on 2020 clinic visits, which may have been limited in many HTCs due to COVID-19, resulting in few participants contributing. A sensitivity analysis of demographic data between the participants included in the ABR and prophylaxis analysis and the total cohort of people with severe hemophilia in the WBDR (*N* = 5084) demonstrated very similar populations, meaning the population analyzed appears to be representative of all people with severe hemophilia in the WBDR. The most notable distinction was in the proportion of pediatric vs adult patients, exhibiting a 3% variance (45% within the examined cohort compared to 48% in the overall group of people with severe hemophilia). Similarly, when considering the distribution of patients among different GNI categories based on ABR, there were differences between the analyzed patients and the total cohort of people with severe hemophilia in the WBDR. Specifically, the percentages differed as follows: 70% vs 66% in LICs and LMICs, 15% vs 24% in UMICs, and 14% vs 10% in HICs. Work productivity was also complicated by several potential confounders, including age, employment availability by country, and the impact of COVID-19 on employment during the time of data collection. These potential confounders were not measured in the WBDR; therefore, the impact on results cannot be quantified. In addition, entering data on work productivity is not mandatory; therefore, the data are based on 67% of patients with these data.

Race and ethnicity are not collected as part of the WBDR, limiting our ability to draw any conclusions based on these variables. Finally, the enrolled participants with severe hemophilia represent a small proportion of the identified and expected populations of people with severe hemophilia [4], limiting generalizability. Low numbers in any given cell, particularly when observing data by GNI and birth cohort, may explain some of the outcome variations, such as the decline in prophylaxis use observed in HICs during the decades 1991 to 2000 and 2011 to 2020. To address cells containing 0 or few patients, the LIC and LMIC categories were combined. Small sample sizes can lead to imprecise estimates and increased variability in the data, which can make it challenging to draw meaningful conclusions.

## 5 Conclusions

The real-world data stemming from the WBDR shows that there have been great advances in care globally for people with hemophilia; however, wide discrepancies between regions of the world still exist, with care in LICs lagging behind higher-income countries by up to 40 years. The data demonstrate not only the negative impact of suboptimal care on patients’ lives but also the positive impact of improvements in care through early diagnosis, access to treatment, and use of prophylaxis.

Today, the WBDR can serve as a model for other rare disease groups in establishing a global registry to track patient outcomes and progress of patient care, identify treatment needs, conduct intercountry comparisons, and support advocacy initiatives with real-world evidence.

## Appendix

World Bleeding Disorders Registry Participating Investigators: Abdel Mohsen M. (Cairo, Egypt), Adeyemo T. (Lagos, Nigeria), Ai Sim G. (George Town, Malaysia), Al-Rahal N. (Baghdad, Iraq), Alexis C. (Bridgetown, Barbados), Ali T. (Damascus, Syria), Awodu O. (Benin, Nigeria), Aysarieva B. (Osh, Kyrgzstan), Aziz A. (Alor Seta, Malaysia), Barsallo N. (Panama City, Panama), Biswas A. (Dhaka, Bangladesh), Blair A. (Winston-Salem, USA), Blatny J. (Czechia), Borhany M. (Karachi, Pakistan), Castillo D. (Havana, Cuba), Catarino C. (Lisbon, Portugal), Chuansumrit A. (Bangkok, Thailand), Coetzee M. (Bloemfontein, South Africa), Darwish Mohamad Ibrahim A. (Mansoura, Egypt), Diop S. (Dakar, Senegal), Djenouni A. (Annaba, Algeria), El Ekiaby A. (Giza, Egypt), El Khorassani M. (Rabat, Morocco), Fawcett K. (Christchurch, New Zealand), Ganieva A. (Osh, Krygzstan), Govindan S. (Manipal, India), Gwarzo D. (Kano, Nigeria), Hailemariam S. (Addis Ababa, Ethiopia), Harper P. (Palmerston North, New Zealand), Hassan T. (Zagazig, Egypt), Hassan M. (Basra, Iraq), Hermans C. (Woluwe-Saint Lambert, Belgium), Hernandez F. (Manila, Philippines), Imran A. (Lahore, Pakistan), John J. (Ludhiana, India), Keikhaei B. (Ahvaz, Iran), Kotila T. (Ibadan, Nigeria), Liam C. (Johor Bahru, Malaysia), Marhaeni W. (Banjarmasin, Indonesia), Mbanya D. (Yaoundé, Cameroon), Mekjarusgul P. (Nakohn Ratchasima, Thailand), Meknassi N. (Rabat, Morocco), Micic D. (Belgrade, Sergia), Mlombe Y. (Lilongwe, Malawi), Motusheva R. (Bishkek, Kyrgzstan), Munube D. (Kampala, Uganda), Nagao A. (Tokyo, Japan), Najmi S. (Kuala Terengganu, Malaysia), Narayana Pillai V. (Aluva, India), Narbekov T. (Bishkek, Kyrgyzstan), Nasution D. (Ampang, Malaysia), Natesirinilkul R. (Chiang Mai, Thailand), Nchimba L. (Lusaka, Zambia), N’dogomo M. (Abidjan, Côte d'Ivoire), Neme D. (Buenos Aires, Argentina), Nguyen P. (Ho Chi Minh City, Vietnam), Nguyen HM. (Hanoi, Vietnam), Nguyen Thi M. (Hanoi, Vietnam), Nigam RK. (Bhopal, India), Njuguna F. (Eldoret, Kenya), Nwagha T. (Enugu State, Nigeria), Obeida A. (Baghdad, Iraq), Owusu-Ofori S. (Kumasi, Ghana), Palascak J. (Cincinnati, USA), Pellegrini G. (Bahia Blanca, Argentina), Philip C. (Tiruvalla, India), Ping CL. (Kuching, Malaysia), Poudyal B. (Kathmandu, Nepal), Rabbani G. (Chittagong, Bangladesh), Rakoto Alson OA. (Antananarivo, Madagascar), Razali H. (Johor Bahru, Malaysia), Ruchutrakul T. (Bangkok, Thailand), Ruiz-Saez A. (Caracas, Venezuela), Saengboon S. (Bangkok, Thailand), Salhi N. (Constantine, Algeria), Satti M. (Khartoum, Sudan), See Guan T. (Seremban, Malaysia), Shah S. (Dhaka, Bangladesh), Shikuku T. (Nairobi, Kenya), Si Yuan N. (Melaka, Malaysia), Sidarthan N. (Kochi, India), Siew Looi T. (Klang, Malaysia), Songthawee N. (Songkla, Thailand), Sosothikul D. (Bangkok, Thailand), Surapolchai P. (Bangkok, Thailand), Suryani S. (Kuala Lumpur, Malaysia), Syakira NA. (Kota Bharu, Malaysia), Thevarajah A. (Kota Kinabalu, Malaysia), Tzong TJ. (Taiping, Malaysia), Udo C. (Abuja, Nigeria), Wong L. (Kota Kinabalu, Malaysia), Yuguda S. (Gombe, Nigeria), Zafar T. (Rawalpindi, Pakistan), and Zaman Miah M. (Rajshahi, Bangladesh).
