# Supplementary material for: The World Federation of Hemophilia World Bleeding Disorders Registry: insights from the first 10,000 patients
Source: Res Pract Thromb Haemost. 2023 Nov 20;7(8):102264. doi: 10.1016/j.rpth.2023.102264 (PMC10772874; doi:10.1016/j.rpth.2023.102264)
Supplement: Supplementary Tables S1–S3 [file mmc1.docx]

**Supplemental Materials**

Table S1: List of data fields in the WBDR dataset

| **Data collected** | **Baseline visit** | **Follow-up visits** |
| --- | --- | --- |
| Date of birth* | X |  |
| Sex* | X |  |
| Country of residence* | X |  |
| Date of diagnosis* | X |  |
| Hemophilia type, severity, factor level at diagnosis* | X |  |
| Bleeding history* | X |  |
| Inhibitor history* | X |  |
| Treatment history* | X |  |
| Blood group | X |  |
| Genetic testing | X |  |
| Bleed assessment (number, location, traumatic/spontaneous) * | X | X |
| Inhibitor assessments* | X | X |
| Target joints* | X | X |
| Hospitalizations* | X | X |
| Treatments* | X | X |
| Adverse events | X | X |
| Co-morbidities | X | X |
| Employment | X | X |
| Mortality* | X | X |
| Self-reported Health-related Quality of life (PROBE, EQ-5D-5L) | X | X |
| Functional scales (HJHS, WFH score (Gilbert) Scale, ROM, FISH and Joint Disease) | X | X |
| COVID-19 | X | X |

*Denotes minimal data set

FISH, Functional Independence Score in Hemophilia; HJHS, Hemophilia Joint Health Score; ROM, range of motion.

Table S2 – Severe hemophilia patients by birth cohort and GNI

|  | **L (N=153)** | **LM (N=3225)** | **UM (N=1207)** | **H (N=499)** | **Total (N=5084)** |
| --- | --- | --- | --- | --- | --- |
| Patients by birth cohort |  |  |  |  |  |
| N | 153 | 3225 | 1207 | 499 | 5084 |
| 1939-1970 | 0 (0%) | 101 (3%) | 48 (4%) | 90 (18%) | 239 (5%) |
| 1971-1980 | 5 (3%) | 172 (5%) | 107 (9%) | 74 (15%) | 358 (7%) |
| 1981-1990 | 18 (12%) | 442 (14%) | 245 (20%) | 81 (16%) | 786 (15%) |
| 1991-2000 | 35 (23%) | 633 (20%) | 282 (23%) | 75 (15%) | 1025 (20%) |
| 2001-2010 | 54 (35%) | 918 (28%) | 301 (25%) | 91 (18%) | 1364 (27%) |
| 2011-2020 | 41 (27%) | 959 (30%) | 224 (19%) | 88 (18%) | 1312 (26%) |

Table S3 - List of participating hemophilia treatment centers (HTCs)

| Algeria - Annaba - Service d’hématologie CHU Annaba |
| --- |
| Algeria - Constantine - Unité hémophilie et maladies hémorragiques héréditaires |
| Argentina - Buenos Aires - Fundación de la Hemofilia and Instituto De Investigaciones Hematológicas “Dr. Mariano R. Castex” |
| Argentina - Bahía Blanca - CARDHE |
| Bangladesh - Dhaka - Bangabandhu Sheikh Mujib Medical University |
| Bangladesh - Rajshahi - Rajshahi Medical College & Hospital |
| Bangladesh - Dhaka - Lab One Foundation |
| Bangladesh - Dhaka - Dhaka Medical College |
| Bangladesh - Chittagong - Chittagong Medical College Hospital |
| Barbados - Bridgetown - Queen Elizabeth Hospital |
| Belgium - Woluwe-Saint-Lambert - Cliniques Universitaires Saint-Luc |
| Cameroon - Yaoundé - CHU Yaoundé |
| Côte d'Ivoire - Abidjan - CHU de Yopougon |
| Cuba - Havana - Instituto de Hematología e Inmunología |
| Czech Republic - IMPORT - HTC |
| Egypt - Mansoura - Mansoura University Children Hospital |
| Egypt - Giza - Shabrawishi Hospital |
| Egypt - Zagazig - pediatrics department, Zagazig University |
| Egypt - Cairo - Pediatric Hemophilia Centre, Ain Shams University |
| Ethiopia - Addis Ababa - Tikur Anbessa Hospital |
| Ghana - Kumasi - Komfo Anokye Teaching Hospital |
| India - Aluva - Haemophilia Treatment Centre, District Hospital |
| India - Bhopal - Gandhi Medical College |
| India - Ludhiana - Christian Medical College |
| India - Manipal - Melaka Manipal Medical College, Hemophilia Society Manipal |
| India - Tiruvalla - Believers Church Medical College Hospital |
| India - Kochi - Amrita Institute of Medical Sciences |
| Indonesia - Banjarmasin - Ulin General Hospital |
| Iran - Ahvaz - Baghaei 2 hospital |
| Iraq - Basra - Basra Center for heriditery Blood Diseases |
| Iraq - Baghdad - National Center of Hematology - Al-Mustansirya University |
| Iraq - Baghdad - Hemophilia Center - Medical City |
| Japan - Tokyo - Ogikubo Hospital |
| Kenya - Nairobi - Kenyatta National Hospital |
| Kenya - Eldoret - Moi Teaching and Referral Hospital |
| Kyrgyzstan - Bishkek - National Center of Oncology and Hematology |
| Kyrgyzstan - Osh - Adult Hematology - Osh Interregional Joint Clinical Hospital |
| Kyrgyzstan - Osh - Dept of Pediatric Hematology - Interregional Children's Clinical Hospital |
| Kyrgyzstan - Bishkek - National Center for Maternity and Childhood |
| Madagascar - Antananarivo - CHU Joseph Ravoahangy Andrianavalona (HJRA) |
| Malawi - Lilongwe - Kamuzu Central Hospital |
| Malaysia - George Town - Hospital Pulau Pinang |
| Malaysia - Ampang - Hospital Ampang |
| Malaysia - Klang - Hospital Tengku Ampuan Rahimah |
| Malaysia - Melaka - Hospital Melaka |
| Malaysia - Kota Kinabalu - Hospital Queen Elizabeth |
| Malaysia - Kuching - Hospital Umum Sarawak |
| Malaysia - Kuala Lumpur - Hospital Kuala Lumpur |
| Malaysia - Johor Bahru - Hospital Sultanah Aminah |
| Malaysia - Kota Bharu - Hospital Raja Perempuan Zainab II |
| Malaysia - Kuala Terengganu - Hospital Sultanah Nur Zahirah |
| Malaysia - Alor Setar - Hospital Sultanah Bahiyah |
| Malaysia - Johor Bahru - Hospital Sultan Ismail |
| Malaysia - Kota Kinabalu - Hospital Wanita dan Kanak-Kanak Sabah |
| Malaysia - Seremban - Hospital Tuanku Ja'afar |
| Malaysia - Taiping - Hospital Taiping |
| Morocco - Rabat - Adultes - Centre de Référence de l'Hémophilie, Hôpital Ibn Sina |
| Morocco - Rabat - Enfants - Centre de Traitement de l'hémophilie de Rabat, Hôpital d'Enfants de Rabat |
| Nepal - Kathmandu - Civil Service Hospital |
| New Zealand - Christchurch - Christchurch Hospital |
| New Zealand - Palmerston North - Palmerston North hospital |
| Nigeria - Kano - Aminu Kano Teaching Hospital |
| Nigeria - Abuja - National Hospital, Abuja |
| Nigeria - Gombe - Gombe State University |
| Nigeria - Enugu State - South East HTC, Department of Haematology, UNTH Ituku Ozalla Enugu |
| Nigeria - Ibadan - University of Ibadan |
| Nigeria - Lagos - Lagos University Teaching Hospital |
| Nigeria - Benin - University of Benin Teaching Hospital |
| Pakistan - Rawalpindi - Haemophilia Treatment Centre |
| Pakistan - Lahore - Haemophilia Treatment Centre |
| Pakistan - Karachi - Haemophilia Welfare Society, Karachi |
| Panama - Panamá City - Hospital del Niño |
| Philippines - Manila - University of Santo Tomas Hospital |
| Portugal - Lisbon - Comprehensive Care Centre of Congenital Coagulopathies, Santa Maria Hospital |
| Senegal - Dakar - Centre National de Transfusion Sanguine |
| Serbia - Belgrade - Mother and Child Health Care Institute of Serbia "Dr Vukan Cupic" |
| South Africa - Bloemfontein - University of the Free State |
| Sudan - Khartoum - Haemophilia Center, Khartoum Teaching Hospital |
| Syria - Damascus - Syrian Hemophilia Society (SHS) |
| Thailand - Bangkok - Department of paediatrics, Ramathibodi Hospital |
| Thailand - Chiang Mai - Chiang Mai University Hospital |
| Thailand - Bangkok - Department of paediatrics, Chulalongkorn University |
| Thailand - Bangkok - Department of medicine, Siriraj Hospital |
| Thailand - Nakohn Ratchasima - Department of paediatrics, Maharat Nakohn Ratchasima Hospital |
| Thailand - Bangkok - Department of paediatrics, Thammasat University |
| Thailand - Bangkok - Department of medicine, Thammasat University |
| Thailand - Songkla - Department of paediatrics, Prince of Songkla University |
| Uganda - Kampala - Mulago Hospital |
| USA - Winston-Salem - Wake Forest Baptist Health |
| USA - Cincinnati - University of Cincinnati Hemophilia Treatment Center |
| Venezuela - Caracas - Centro Nacional de Hemofilia - Banco Municipal de Sangre DC |
| Vietnam - Hanoi - National Institute of Hematology and Blood Transfusion |
| Vietnam - Ho Chi Minh City - Blood Transfusion Hematology |
| Vietnam - Hanoi - National Children's Hospital |
| Zambia - Lusaka - University Teaching Hospital |
